# Identifying the Novel Inhibitors Against the Mycolic Acid Biosynthesis Pathway Target “mtFabH” of *Mycobacterium tuberculosis*

**DOI:** 10.3389/fmicb.2022.818714

**Published:** 2022-05-06

**Authors:** Niranjan Kumar, Rakesh Srivastava, Raj Kumar Mongre, Chandra Bhushan Mishra, Amit Kumar, Rosy Khatoon, Atanu Banerjee, Md Ashraf-Uz-Zaman, Harpreet Singh, Andrew M. Lynn, Myeong-Sok Lee, Amresh Prakash

**Affiliations:** ^1^School of Computational and Integrative Sciences, Jawaharlal Nehru University, New Delhi, India; ^2^Molecular Cancer Biology Laboratory, Cellular Heterogeneity Research Center, Department of Biosystem, Sookmyung Women’s University, Seoul, South Korea; ^3^Department of Microbiology and Immunology, David H. Smith Center for Vaccine Biology and Immunology, University of Rochester Medical Center, Rochester, NY, United States; ^4^Department of Pharmacology and Chemical Biology, Baylor College of Medicine, Houston, TX, United States; ^5^Indian Council of Medical Research–Computational Genomics Centre, All India Institute of Medical Research, New Delhi, India; ^6^Amity Institute of Integrative Sciences and Health, Amity University, Gurugram, India; ^7^Amity Institute of Biotechnology, Amity University, Gurugram, India

**Keywords:** *Mycobacterium tuberculosis*, mycolic acids, mtFabH, inhibitor, MD simulation, MM/PBSA

## Abstract

Mycolic acids are the key constituents of mycobacterial cell wall, which protect the bacteria from antibiotic susceptibility, helping to subvert and escape from the host immune system. Thus, the enzymes involved in regulating and biosynthesis of mycolic acids can be explored as potential drug targets to kill *Mycobacterium tuberculosis* (Mtb). Herein, Kyoto Encyclopedia of Genes and Genomes is used to understand the fatty acid metabolism signaling pathway and integrative computational approach to identify the novel lead molecules against the mtFabH (β-ketoacyl-acyl carrier protein synthase III), the key regulatory enzyme of the mycolic acid pathway. The structure-based virtual screening of antimycobacterial compounds from ChEMBL library against mtFabH results in the selection of 10 lead molecules. Molecular binding and drug-likeness properties of lead molecules compared with mtFabH inhibitor suggest that only two compounds, ChEMBL414848 (C1) and ChEMBL363794 (C2), may be explored as potential lead molecules. However, the spatial stability and binding free energy estimation of thiolactomycin (TLM) and compounds C1 and C2 with mtFabH using molecular dynamics simulation, followed by molecular mechanics Poisson–Boltzmann surface area (MM/PBSA) indicate the better activity of C2 (ΔG = −14.18 kcal/mol) as compared with TLM (ΔG = −9.21 kcal/mol) and C1 (ΔG = −13.50 kcal/mol). Thus, compound C1 may be explored as promising drug candidate for the structure-based drug designing of mtFabH inhibitors in the therapy of Mtb.

## Introduction

*Mycobacterium tuberculosis* (Mtb) dodges successfully the host immune clearance by encapsulation, specifically with mycolic acids, which are resistant to usual macrophage degradation procedures. The cell wall mycolic acid encapsulation further protects Mtb from the antibiotic actions. Thus, the mycolate biosynthesis is an excellent drug target to kill the *Mycobacterium*. Two distinct but related *de novo* synthesis of fatty acid systems (FASs), which perform biosynthesis of fatty acid, importantly involve in the cell wall development and maintenance (Molle et al., 2006). Eukaryotes use a big multifunctional protein of single or double polypeptides chains (type I FAS approach) (Wakil, 1989; Schweizer and Hofmann, 2004), whereas the protozoan, prokaryote, and plant type II FAS approach consists of distinct enzymes with monofunctional activities that correspond to type I single-chain FAS systems (Rock and Cronan, 1996; Surolia and Surolia, 2001).

In Mtb, the β-ketoacyl-acyl carrier protein (ACP) synthase III enzyme is known as mtFabH and is the key connecting link between FAS-I and FAS-II pathways of mycolic acid biosynthesis. Sequential elongation steps catalyzed by multifunctional (type I) fatty acid synthase complex assisted by dissociated (type II) fatty acid synthase, which is formed by long-chain α-alkyl-β-hydroxy long fatty acids, called mycolic acids, which are typical elements of the *Mycobacterium* cell wall (Heath et al., 2001; Wang et al., 2006). The first step in elongation reaction in bacterial type II fatty acid synthesis is the Claisen condensation of acetyl-CoA with a malonyl-ACP catalyzed by β-ketoacyl-ACP-synthase III (FabH) (Choi et al., 2000).

The elongation of FAS-II takes part in the synthesis of mycolic acids, which are unique and fundamental fatty acids long-chain of the Mtb cell wall and other species of *Mycobacterium* (Asselineau et al., 2002), including *Mycobacterium smegmatis*. Mycobacterium FabH is a type II fatty acid synthase enzyme that forms long-chain (C16–C22) acyl-coenzyme A (CoA) and mycolic acids precursors, which are key components of the bacterial cell wall (Sachdeva and Reynolds, 2008). The product C16 acyl-CoA serves as a precursor for FAS-II meromycolic acid synthesis, whereas the fatty acid C26 represents the α branch of the last mycolic acid. Studies suggest that mtFabH is the connecting link between FAS-I and FAS-II by translating FAS-I produced C14-CoA to C16-AcpM, which is channeled into the FAS-II cycle (Bhatt et al., 2007). Unlike the enzymes in FAS-I, FAS-II enzymes, like mtFabH, are also not present in mammals, which suggest the excellent drug target for the development of Mtb inhibitors. Thiolactomycin (TLM), the existing antituberculosis drug against mtFabH targeting mycolic acid biosynthesis, was primarily obtained from the bacteria actinomycete *Nocardia* species. It is a specific compound exhibiting selective action only against the dissociable site of type II fatty acid synthase (FAS-II). TLM specifically inhibits Mtb FAS-II by targeting *in vivo* and *in vitro* β-ketoacyl-ACP-synthase (mtFabH and mtFabB), contributing to inhibition of mycolic acid biosynthesis in the cell wall and results in cell death (Schaeffer et al., 2001; Kremer et al., 2002). Although much is being gained from such structural based rational designing of drug molecules, which can be an incredible approach for the development of novel inhibitors against mtFabH (Brown et al., 2009; Das et al., 2016; Mongre et al., 2019; Atale et al., 2021; Saha et al., 2021).

The present study incorporates the fatty acid metabolism signaling pathway analysis applying Kyoto Encyclopedia of Genes and Genomes (KEGG), identification of potential molecular target mtFabH, crucial for the biosynthesis of Mtb cell wall. Glide is a reliable molecular docking tool that provides three-step precision filtering, used for virtual screening of antimycobacterial compounds from ChEMBL. The binding affinity and pharmacokinetic profile analyses of lead molecules and mtFabH inhibitor TLM indicate the promising activity of ChEMBL compounds, ChEMBL414848 (C1), and ChEMBL363794 (C2). However, the molecular stability and binding free energy estimation using MD simulation and molecular mechanics Poisson-Boltzmann surface area (MM/PBSA) suggest the more promising activity of compound C2 as compared with TLM and C1. Thus, this study provides the structural insight for the further development of potential mtFabH inhibitors in the eradication of Mtb.

## Materials and Methods

### From Drug Target to Molecular Network

Network analyses were performed to identify the molecular targets involved in the biosynthesis of mtFabH using KEGG. It involves the comparable analysis of pathways, which can be used to demonstrate the key elements, interacting to the various pathways. KEGG Mapper was applied for mapping the pathways that are being used popularly for understanding of biological genome sequences and other high-throughput biological data. The pathways of KEGGs are represented graphically in which nodes represent genes, enzymes, or compounds, and edges encode relationship, reaction, or node interactions. The three databases, BRITE, PATHWAY, and MODULE, comprise new information collected from published literature of such high-level functions and described in terms of BRITE hierarchies, KEGG pathway, and KEGG modules, respectively, usually known as KEGG molecular networks; they reflect certain elements of the network of molecular interaction, reaction, and relationship (Kanehisa et al., 2016). The KEGG molecular networks are generally formed, such as based on a functional orthology group named KO (KEGG Orthology) instead of particular genes or protein, so that experimentation information can be applied to other species in particular organisms. This can be important in assessing regulatory events affecting various biological functions and pathways (Kanehisa and Sato, 2020).

### Molecular Docking Analysis

Using the GLIDE (drift-based ligand docking with energetic) module of Schrödinger, suite-2019-1, the molecular docking of ligands was performed with the crystal structure of mtFabH (PDB ID: 1HZP). The protein was prepared by removing water molecules and other heteroatoms, lauric acid, and glycerol. The non-polar hydrogen atoms were added followed by the minimizing energy. Schrodinger suite’s LigPrep module 2019-1 was used for the ligands preparations. Applying the OPLS 2005 force field, it was allowed to generate the maximum 32 conformational for each compound. The binding site of protein was chosen as described in the cocrystallized structure of mtFabH with inhibitor (Scarsdale et al., 2001a). The active site for molecular docking was defined around 10Å, considering the catalytic triad amino acid residues, Cys112, His244, and Asn274. The grid was created over the active site, and ligands were allowed for flexibly docking, and the non-planar conformations were penalized (Prakash and Luthra, 2012; Mishra et al., 2018; Mishra et al., 2020a). Finally, the top-ranked 10 compounds were selected based on the extra precision (XP) Glide scores (gscores) and docking score. PyMOL (Mooers, 2016) was used to illustrate the molecular interactions of ligands with mtFabH and the two-dimensional interactions with the contributing residues stabilizing the ligands at active site were drawn by LigPlot+ v1.4.5 (Wallace et al., 1995).

### Pharmacokinetics (Absorption, Distribution, Metabolism, Excretion, and Toxicity) Analysis

The top 10 compounds showing the best docking score were taken for the pharmacokinetic profile and drug-likeness (DL) analyses (Thangarasu et al., 2018). The ADME (absorption, distribution, metabolism, and excretion) analyses were performed using admetSAR (Cheng et al., 2012), and the toxicity of the compounds was predicated using the OSIRIS Property Explorer (Sander et al., 2009).

### Molecular Dynamics Simulation

Molecular dynamics (MD) simulation was performed using the Amber16 biosimulation package on the coordinates of mtFabH and mtFabH–ligand complexes. The force field was selected as ff14SB, and TIP3P water model was applied for the solvation of prepared systems. Using Antechamber tool, the force field GAFF and AM1-BCC charges were applied to define the parameters and topology for ligands. Four separate MD runs have been carried out for the prepared systems, mtFabH, mtFabH-TLM, mtFabH-CHEMBL414848, and mtFabH-CHEMBL3637. The models were planned in the octahedral simulation box employing Amber16 tleap method with buffer distance (12Å); 0.15 M counter ions (Na^+^ and Cl^–^) were added to neutralize the system. Hydrogen bonds (H-bonds) were treated with the SHAKE algorithm, and electrostatic long-range forces were controlled using the Ewald summation of particle mesh (Joung and Cheatham, 2008). Berendsons barostat and Langevin thermostat were used and applied during the simulation to maintain the temperature and pressure, respectively. Energy minimization was performed in two steps. The first phase should include 3,000 steps of minimization, which implicated 2,500 steps of the steepest gradient and the remaining 500 steps by conjugate gradient algorithm. Solute atoms (100 kcal mol^–1^Å^–2^) were limited, and only water molecules and counter ions were permitted to pass. The second minimization process comprised 5,000 steps of minimization (conjugate gradient: 500 and steepest gradient: 4,500 steps used) with no restrictions on every atom. Minimization phase preceded by equilibrium of the heat exchanger from 0 to 298 K with a sampling interval of 1-fs for 30-ps and a 100-ps simultaneous balance run using a 2-fs time stage with NPT ensemble. The output run was executed for duration of 100 ns on NPT ensemble using pmemd.cuda, and the time stage was set to 2 fs. At the gap of 10 ps, all data, trajectories, speed, and energy were saved. The trajectories of the simulation were analyzed using the Amber16 cpptraj tool (Pandey et al., 2020; Niranjan et al., 2021).

### Essential Dynamics Analysis

Essential dynamics (ED) is a statistical method with multivariate. Systematically, it is implemented to minimize the number of dimensions required to explain the dynamics of proteins. Systematically decreasing the number of dimensions is known as decomposition method. ED filters identified movements from the largest to the smallest spatial scales (David and Jacobs, 2014). The overall protein extension during the various simulations is studied using the ED or principal component analysis (PCA) (Maisuradze et al., 2009). ED is the method of implementing PCA to trajectories of a protein or protein–ligand complex because the “essential” motion is derived from the studied conformation set (David and Jacobs, 2014; Mishra et al., 2020a). The amount of the eigenvalue is equal to the absolute motility in the system. ED is an essential study, which can measure a protein’s flexibility among various conditions (Tiana et al., 2004). Moreover, the ED method is used to collect important and specific biological motions from global trajectories.

### Binding Free Energy Calculation

The strength of protein–ligand binding was analyzed by estimating the binding free energy. We have utilized an implicit solvation model approach for the binding free energy calculation. There are two popular methodologies considering the implicit nature of the solvent for binding free energy calculation: MM/PBSA (Genheden and Ryde, 2015). In this method, the protein and ligand are taken in a detailed all-atom model while the solvent is modeled as a dielectric continuum. The atom-atom interactions are estimated through molecular mechanics force field parameters while solvent electrostatics is determined using the Poisson–Boltzmann equation. Other non-polar solvent contributions are estimated by calculating the surface area of the molecules (Miller et al., 2012). The interior of the protein molecule was modeled with a dielectric constant of 4, and solvent dielectric constant was taken as 80. The rest of the parameters were kept default. The free energy of a molecule in a solvent can be approximately written as follows:


$$G=E_{\text{MM}}+G_{\text{sol}}+T\,S$$

where *E*_*MM*_, *G*_*sol*_, and *TS* are the molecular mechanics force field energy, solvation free energy, and entropic contribution (*T*: absolute temperature and *S*: entropy). Thus, the binding free energy of a protein–ligand system can be written as follows:


$$△\,G_{\text{bind}}=△\,E_{\text{MM}}+△\,G_{\text{sol}}+T\,△\,S$$

Neglecting the entropic contribution, which is usually quite small, the binding free energy can be written as follows:


$$△\,G_{\text{bind}}=△\,E_{\text{MM}}+△\,G_{\text{sol}}$$

The first term on the right side can be estimated using molecular mechanics force field, and the second term using a proper solvation model (generalized Born or Poisson–Boltzmann). We have used 500 equally spaced frames from the last 50-ns simulation data to calculate the binding free energy.

## Results and Discussion

### Molecular Pathway Analysis

**FIGURE 1:**
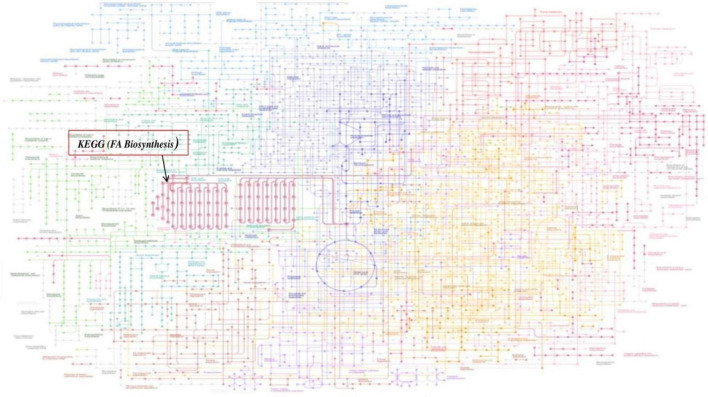
Global metabolic pathways of fatty acid (FA) biosynthesis using KEGG Mapper. The *Mycobacterium* bacterial metabolic pathways are colored, and the fatty acid (FA) pathways are in red indicated with arrow.

The enzyme β-ketoacyl ACP synthase (KASIII or FabH) is the key enzyme that initiates fatty acid biosynthesis in a type II dissociated FAS. This enzyme catalyzes the condensation of acyl CoA and malonyl ACP to form a β-ketoacyl ACP product, which is further processed to form mature fatty acids that are involved in various essential cellular processes and structures such as phospholipid biosynthesis, cell wall formation, and so on. KEGG Mapper was applied to link the differentially expressed genes involved in the signaling pathways of fatty acid biosynthesis, regulated by FabH in Mtb. Results show the effect of FabH in the metabolic pathways FA biosynthesis. KEGG Mapper used for inferring the cellular functions from protein sequences shows that the enzyme FabH is involved in regulating many essential metabolic processes in bacteria such as pyruvate metabolism, β-alanine metabolism, lipoic acid metabolism, fatty acid degradation, glycolipid metabolism, glycerophospholipid metabolism, and fatty acid elongation, which can be explored as potential molecular targets in antibacterial drug discovery. KEGG Mapper consists of BRITE hierarchies, KEGG modules, and KEGG pathway to analyze the result of many signaling pathways, and the number in parentheses after the pathway name is the number of proteins in the pathway of our interest. Figure 1 shows the general global integrated maps of metabolic pathways, and the arrow indicates fatty acid pathways in bacteria.

**FIGURE 2:**
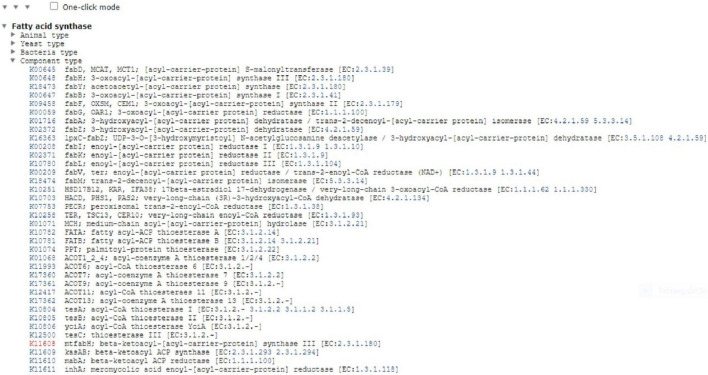
Fatty acid synthase hierarchical classification studies from BRITE database.

Furthermore, the BRITE database explored which performs a function in the classification of chemical compounds and reactions, genes and proteins, diseases, and organisms. FabH classification unit in bacteria is described in Figure 2. It explained the tree-like structure of fatty acid synthase, which was defined as a hierarchical file system, as displayed in the classification. However, our interest is component type K11608, FabH which belongs to Mtb.

**FIGURE 3:**
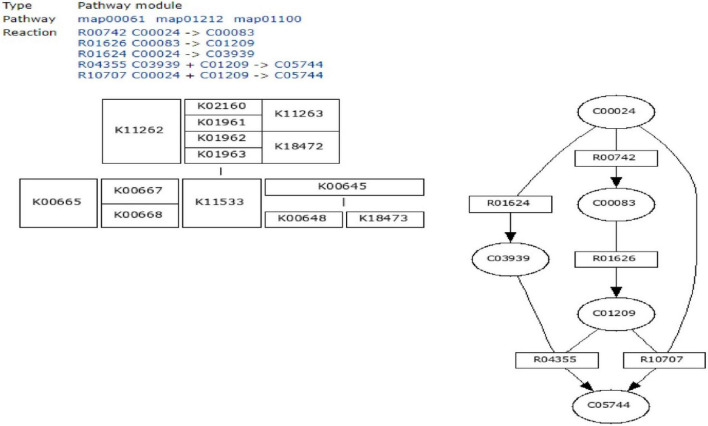
Initiation of fatty acid biosynthesis pathway using MODULES.

**FIGURE 4:**
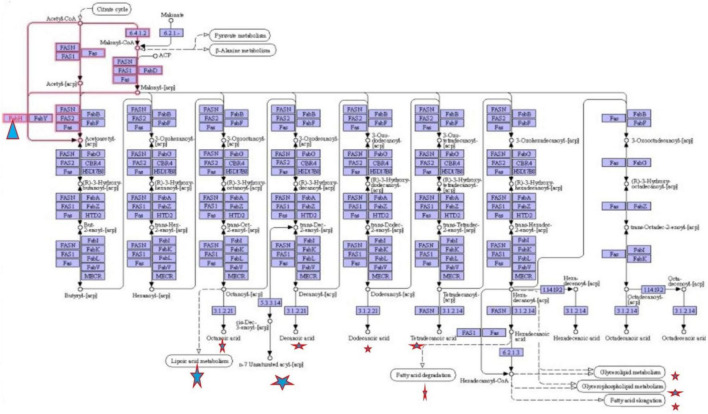
Fatty acid biosynthesis different pathways regulating by FabH.

To define the functional units, reactions, and signature, KEGG modules were explored for determining the logical expression; space or a plus sign, implying a relationship in the molecular complex or pathway, is known as the AND operator, and the comma used for alternatives, which is called OR operator; analysis of signaling pathways is shown in Figures 3, 4.

Final study revealed the fatty acid metabolism signaling pathway and pathway map of mycolic acid biosynthesis, which explains that FabH interacts with several other metabolism pathway and affects bacterial cell wall biosynthesis such as fatty acid degradation, fatty acid elongation, and lipoic acid metabolism, which is shown in Figure 4.

### High-Throughput Virtual Screening

A series of antimycobacterial compounds from the ChEMBL library were taken for the virtual screening against the drug target mtFabH. The inhibitor TLM was used as a reference compound to define the protein active site and compare the molecular interactions with lead molecules. ChEMBL consists of the bioactive conformers of 78,713 compounds, having antimycobacterial activity (Kumar et al., 2020; Niranjan et al., 2021). Applying Lipinski’s rule of five for filtering the DL, only 4,754 compounds were passed successfully. Using the Glide docking protocol, having three-step filtering methods: high-throughput virtual screening, SP, and XP results in the selection of top 10%, 45 compounds. Finally, the best 10 ranked ligands were selected for ADMET (adsorptions, distributions, metabolism, excretion, and toxicity). The molecular interaction of inhibitor, TLM, with mtFabH shows the docking score of −3.73 kcal/mol, whereas compounds from the ChEMBL database represented a wide range of docking scores: −6.34 to −8.51 kcal/mol. Among the best 10 hit compounds, C1 shows the highest docking score −8.51 kcal/mol. The docking score and Glide XP binding energy top 10 hit molecules with control inhibitors, TLM, are summarized in Table 1.

**TABLE 1:** Molecular docking of docking and post-docking analysis of CHEMBL (bioactive) antituberculosis compounds against mtFabH.

S. No.	Compounds	Docking score	HBI	Hydrophobic interaction
01	Thiolactomycin (TLM)	–3.73	Arg249	Trp32, Arg36, Thr37, Phe155, Ile156, Leu207, Gly209, Pro210, Val212, Asn247.
02	CHEMBL414848 (C1)	–8.512	Asn274 Tyr304	Trp32, Arg36, Cys112, Phe155, Ile165, Ile189, Leu207, Gly209, Val212, Phe213, His244, Ala246, Asn247, Gly305.
03	CHEMBL363794 (C2)	–7.246	Asn274	Cys112, Ile156, Ile189, Leu207, Gly209, Pro210, Val212, Phe213, Ala246, Asn247, Arg249, Gly305.
04	CHEMBL549989 (C3)	–7.219	Asn274	Trp32, Gly152, Phe155, Ile156, Leu207, Gly209, Pro210, Val212, Phe213, Ala246, Asn247, Ala249, Ile250.
05	CHEMBL565500 (C4)	–7.081	Asn274	Trp32, Arg36, Thr37, Ile156, Ile189, Leu207, Val212, Phe213, Ala246, Asn247, Ile250.
06	CHEMBL515441 (C5)	–6.713	Asn247 Asn274	Trp32, Arg36, Thr37, Ile156, Phe157, Ile189, Leu207, Gly209, Pro210, Val212, Phe213, His244, Ala246, Asn247, Tyr304, Gly305.
07	CHEMBL475041 (C6)	–6.522	Asn247 Asn274	Trp32, Arg36, Thr37, Phe155, Ile156, Phe157, Ile189, Leu207, Gly209, Val212, Phe213, His244, Ala246, Arg249, Tyr304, Gly305.
08	CHEMBL474052 (C7)	–6.481	Asn247 Asn274	Trp32, Arg36, Thr37, Phe155, Ile156, Phe157, Ile189, Leu207, Gly209, Val212, Phe213, His244, Ala246, Arg249, Tyr304, Gly305.
09	CHEMBL475851 (C8)	–6.448	Asn274	Trp32, Arg36, Thr37, Phe155, Ile156, Phe157, Ile189, Leu207, Gly209, Val212, Phe213, His244, Ala246, Arg249, Tyr304, Gly305.
10	CHEMBL572316 (C9)	–6.437	Arg249	Gly152, Phe155, Ile156, Leu207, Gly209, Val212, Phe213, Arg214, Ala246, Asn247, Ile250.
11	CHEMBL495223 (C10)	–6.348	Asn274	Thr37, Gly152, Phe155, Ile156, Leu207, Gly209, Val212, Phe213, Ala246, Asn247, Ile250.

### Absorption, Distribution, Metabolism, Excretion, and Toxicity Analysis

In the process of drug development, the successful examination of pharmacokinetic properties is the essential steps for selection of potential compounds (Davis and Riley, 2004). This process reduces the time and cost as well as the chance to be eliminated in the clinical trials (Tang et al., 2006). Earlier studies suggest the potential inhibitory activity of TLM and platensimycin against the mtFabH. However, because of poor pharmacokinetic properties and negligible oral bioavailability, several efforts are ongoing to develop the analogous compounds with improved pharmacokinetic profiles (Brown et al., 2009; Das et al., 2016). After the screening and docking, the identified best hit 10 compounds are further analyzed for ADME and toxicity and compared with mtFabH inhibitor, TLM. The ADME results include cLogP (lipophilicity), renal organic cation transporter (ROCT), cytochrome P450 (CYP450 2D6), human intestinal absorption (HIA), blood–brain barrier (BBB), aqueous solubility (logS), and topological polar surface area (TPSA), which are summarized in Table 2. In the predicted HIA, positive sign (HIA+) indicates good intestinal absorption, and the negative sign (HIA−) represents poor intestinal absorption. The compounds that could be able to cross the BBB were shown as BBB+, and those that could not be able to cross the BBB were represented as BBB−. The ROCT is another important parameter that predicted the uptake or efflux of cationic drugs. Results display that the control inhibitor, TLM, passes all the examined properties, for example, HIA, BBB, ROCT, logS, CYP450 2D6 inhibitor, cLogP (hydrophilicity), and TPSA. Similarly, except the lead compounds (C3) CHEMBL549989 and (C10) CHEMBL495223, all others showed better BBB score. The logS results show better scores for compounds, (C1) CHEMBL414848, (C2) CHEMBL363794, (C3) CHEMBL549989, (C4) CHEMBL565500, (C9) CHEMBL572316, and (C10) CHEMBL495223. The good scores recommended for TPSA are between ≤90 and 100Å^2^; except C9 and C10, all compounds show better TPSA scores. Thus, the overall analysis of ADME results suggests that only C1, C2, and C3 compounds may be explored as potential lead molecules.

**TABLE 2:** ADME analysis of mtFabH inhibitor and lead molecules.

S. No.	Compounds	HIA	BBB	ROCT	CYP4502D6 inhibitor	Solubility (logS)	ClogP	TPSA (Å^2^)
1	TLM	HIA+	BBB+	NI	NI	–2.267	2.720	62.60
2	C1	HIA+	BBB+	NI	NI	–2.554	0.490	92.50
3	C2	HIA+	BBB+	NI	NI	–2.920	0.884	86.71
4	C3	HIA+	BBB–	NI	NI	–3.747	3.604	66.63
5	C4	HIA+	BBB+	NI	NI	–3.900	3.552	52.33
6	C5	HIA+	BBB+	NI	NI	–4.161	3.265	74.45
7	C6	HIA+	BBB+	NI	NI	–4.143	3.407	74.45
8	C7	HIA+	BBB+	NI	NI	–4.359	3.669	74.45
9	C8	HIA+	BBB+	NI	NI	–4.081	3.063	74.45
10	C9	HIA+	BBB+	NI	NI	–3.482	3.036	105.80
11	C10	HIA+	BBB-	NI	NI	–1.484	–0.645	106.80

### Toxicity Analysis

Osiris (Sander et al., 2009; Singhal and Saxena, 2015) and admetSAR (Cheng et al., 2012) are the excellent examples of open-source computational tools for the comprehensive analyses of compounds toxicity. Osiris was used to examine the tumorigenicity (TUM), and AMES test for the probable mutagen, cancer-causing agents by mutagenic effect analysis, reproductive risk, irritant, and DL. The toxicity analysis results of TLM and ChEMBL lead molecules are shown in Table 3. A positive value serves as a lead molecule for future development of antimycobacterial tuberculosis commercial drugs.

**TABLE 3:** Toxicity analysis of known inhibitor TLM and lead molecules.

S. No.	Compounds	TUM	AMES toxicity	Mutagenic	Reproductive effect	Irritant	DL
1	TLM	No	No	No	No risk	No	–1.776
2	C1	No	No	No	No risk	No	4.410
3	C2	No	No	No	High-risk	No	5.799
4	C3	No	No	No	No risk	No	–9.087
5	C4	No	No	No	No risk	No	–11.269
6	C5	No	No	No	No risk	No	–9.211
7	C6	Yes	No	No	Yes	Yes	–8.611
8	C7	No	No	No	No risk	No	–8.555
9	C8	No	No	No	No risk	No	–9.262
10	C9	No	Yes	No	No risk	No	–7.121
11	C10	No	No	No	No risk	No	–1.213

Results revealed that TLM passes in all toxicity analyses, which include TUM, AMES toxicity, mutagenic effect, reproductive toxicity effect, and irritant; however, a negative value was obtained for DL property. Usually, the positive values for DL property are favorable, but several drugs and fluka chemicals have negative values in medication. All selected lead molecules show no mutagenic effect, except compound C6, which is predicted as tumorigenic as well as irritant. The compounds C9 shows AMES toxicity property, and C6 is associated with reproductive toxicity. Among all compounds, only two compounds, C1 and C2, show a good score for DL analysis, which suggests acceptable pharmacokinetic properties. Thus, for the docked complexes of mtFabH with TLM, compounds C1 and C2 are selected for further analysis, applying MD simulation, followed by the binding free energy estimation using MM-PBSA.

### Molecular Interactions

**FIGURE 5:**
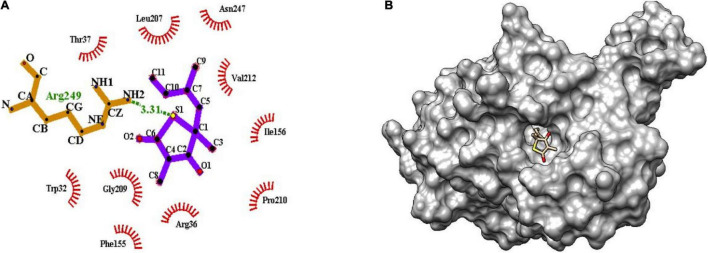
**(A)** Molecular interaction of inhibitor TLM at the active site of mtFabH using LigPlot. **(B)** Surface view of mtFabH-TLM complex using Schrodinger.

**FIGURE 6:**
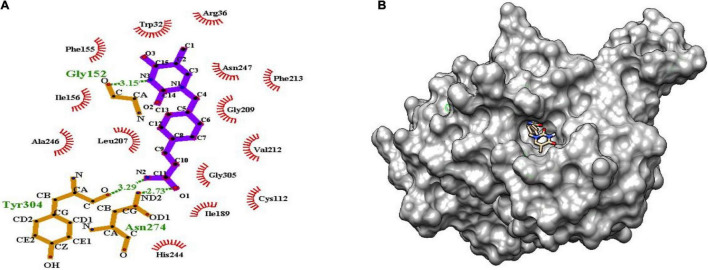
**(A)** Molecular interaction of lead molecule, ChEMBL414848 (compound C1) at the active site of mtFabH using LigPlot. **(B)** Surface view of mtFabH-ChEMBL414848 complex.

**FIGURE 7:**
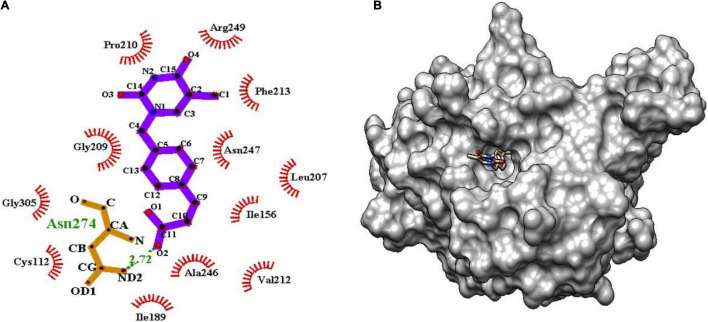
**(A)** Molecular interaction of lead molecule, ChEMBL363794 (compound C2) at the active site of mtFabH using LigPlot. **(B)** Surface view of mtFabH-ChEMBL363794 complex.

**FIGURE 8:**
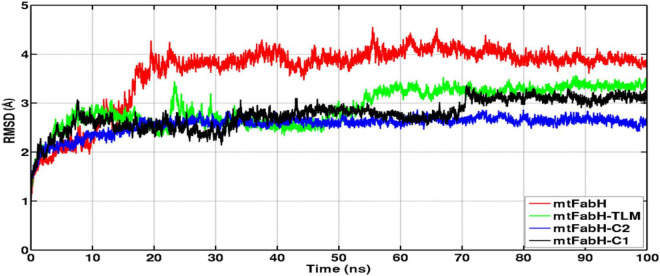
Time evolution plots of Cα-backbone RMSD of mtFabH (red), mtFabH-TLM complex (green), mtFabH-C2 (blue), and mtFabH-C1 (black). The MD trajectories were plotted using xmgrace.

The binding of inhibitor at the active site of mtFabH shows that TLM is spatially stabilized by H-bond interaction with Arg249, whereas residues Thr37, Leu207, Asn247, Val212, Ile156, Pro210, Arg36, Phe155, Gly209, and Trp32 are involved in hydrophobic interactions as shown in Figure 5. The side chain of TLM, (5R)-4-hydroxy-3, 5-dimethyl-5-[(1E)-2-methylbuta-1,3-dienyl] thiophen-2-one at C5 position maintains conjugated-double bonds to build hydrophobic interactions Thr37, Ile156, Leu207, Asn247, and Val212. Similarly, the C3 methyl group interacted with hydrophobic interaction residues Trp32, Arg36, Phe155, and Gly209, whereas the thiophen group of TLM possesses one H-bond between the S1 positions of thiolactone component placed in the surface of the active site with the H-bond distance of 3.31Å to NH2 group of Arg249. The lead compounds are involved in H-bond interaction with residues Asn247, Arg249, Asn274, and Tyr304 and the hydrophobic interactions with residues Trp32, Arg36, Thr37, Cys112, Gly152, Phe155, Ile156, Phe157, Ile189, Leu207, Gly209, Pro210, Val212, Phe213, His244, Ala246, Asn247, Arg249, Ile250, Tyr304, and Gly305, as enumerated in Table 2, Figures 6–8, and Supplementary Figure 1.

Figure 6 shows that the dioxo pyrimidine ring of compound C1 (E)-3-[4-[(5-methyl-2,4-dioxopyrrolidin-1-yl) methyl] phenyl] prop-2-enamide is involved in hydrophobic interaction with residues Trp32, Arg36, Phe155, Ile156, Leu207, Gly209, Val212, Phe213, and Ala246. The active site residue Asn247 is involved in H-bond interaction (3.15Å) with N3 atoms of the pyrimidine and carboxylate group of residues Gly152. The 2-propene group of C10 atoms interacts with residues Cys112 and Gly305 and amide group of O1 atoms between ND2 atoms linked by H-bond with residue Asn274 (2.73Å). Similarly, the amide group of N2 atoms and hydroxyl group of O atoms are involved in H-bond interactions (3.29Å) with residue Tyr304. These interactions were observed to be consistent with the molecular interactions of TLM at the active site of mtFabH (Scarsdale et al., 2001b).

Similarly, the second hit best compound **C2**, (E)-3-[4-[(5-methyl-2,4-dioxopyrimidin-1yl) methyl] phenyl] prop-2-enoic acid shows hydrophobic interactions with residue Ile156, Leu207, Pro210, Phe213, and Arg249. The pyrimidine moiety of ligand stabilized by hydrophobic interactions and phenyl group is oriented toward residues Ile156, Leu207, and Asn247. The propene group also formed hydrophobic interaction Cys112, Ile189, Val212, Ala246, and Gly305 and H-bond interaction with Asn274 (2.72Å) as shown in Figure 7.

### Molecular Dynamics Simulation

The efficacy of a drug molecule relies on the effective binding at active site of target protein and the conformational stability of protein–ligand complex (Luthra et al., 2009; Mishra et al., 2018; Majewski et al., 2019; Wang et al., 2020). The structure-based virtual screening is one of the reliable strategies to identify a potential inhibitor in the drug development process (Meng et al., 2011). But the major challenge for existing docking tools is to deal with the flexibility of protein for which several efforts are ongoing (Forli et al., 2016; Sulimov et al., 2019; Mishra et al., 2020b). Thus, to understand the protein–ligand molecular interactions, MD simulation can provide an atomistic insight on the structural stability and dynamic of protein–ligand interactions (Prakash and Luthra, 2012; Panda et al., 2020). Based on the virtual screening and extensive ADMET analysis results in the selection of two ChEMBL compounds, C1 and C2 show promising activities and may be explored as plausible mtFabH inhibitors. Thus, to examine the conformational stability and dynamics of protein–ligand complexes, multiple MD simulations were performed for the period of 100 ns at the physiological temperature of 300 K.

The RMSD of protein backbone measures the deviation in protein structure with respect to the initial structure and thus gives an idea about global protein flexibility. The C^α^-backbone RMSD plots of mtFabH complexed with TLM, C1, and C2 are shown in Figure 8.

Results show that the RMSD trajectories of four systems, mtFabH, and complexed with ligands converge around 20 ns, and a stable equilibrium remains consistent for the remaining period of simulation, 20–100 ns. Although the docked complex of mtFabH-TLM quickly achieved the equilibrium at ∼5.0 ns and remained stable up to 50 ns, showing the minor perturbation at ∼20–30 ns. The consecutive increase in RMSD can be seen at ∼50–60 ns, thereafter the trajectory observed was stable until 100 ns. Remarkably, the RMSD trajectory of mtFabH-C2 attains equilibrium at ∼15 ns, and a steady equilibrium can be seen throughout the simulation time of 15–100 ns. The complex of mtFabH-C2 shows that it readily achieved the equilibrium at ∼8 ns, which remains consistent up to 30 ns. With the small drift of approximately 1.0Å, RMSD value increased to 2.5Å; however, the trajectory converges smoothly, and a stable equilibrium can be seen for the period of ∼35–70 ns. Because of sharp drift of ∼1.0Å, the rise in RMSD is seen, but it settles promptly, and a well-stabilized conformational dynamics with the average RMSD value ∼3.0Å is observed during the 70–100 ns.

**FIGURE 9:**
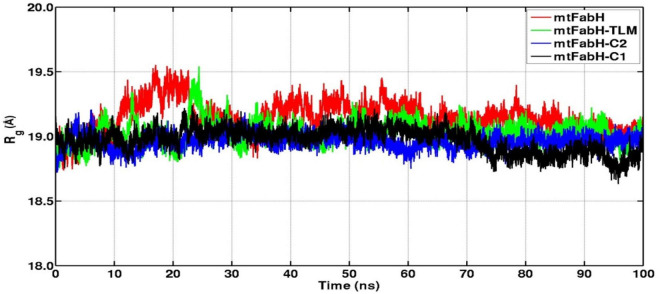
Radius of gyration (Rg) plots of mtFabH and complexed with TLM and lead molecules, C1 and C2.

We further analyzed the conformational dynamics using the radius of gyration (Rg), which measures the protein structural compactness. Figure 9 indicates that the structural dynamics of mtFabH remains confined with average Rg value 19.30 ± 0.20Å. The drifts in trajectory can be seen at 10–20 ns, which settled at ∼30 ns, and a consistent structure is prolonged until the simulation finished at 100 ns. The Rg plot of TLM shows consecutive drifts during the initial, which optimized at ∼35 ns, and the structural dynamics was observed to be stable with Rg value 19.20 ± 0.30Å. The plot of mtFabH-C1 shows rather no changes or minimal perturbation, which can be observed throughout the simulation period of 0–100 ns. The structure remains confined to Rg value 19.10 ± 0.10Å that suggests a stable binding of compound C1 with mtFabH. Similarly, except the initial agitation in Rg trajectory of mtFabH-C2, no major deviations are observed during 25–100 ns, and the remaining ones stabilized with Rg value 19.20 ± 0.20Å.

**FIGURE 10:**
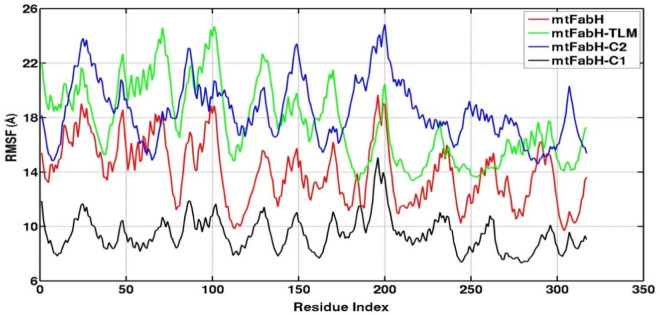
Root mean square fluctuation (RMSF) plots of mtFabH and docked complexes with ligands, TLM, C2, and C1.

**FIGURE 11:**
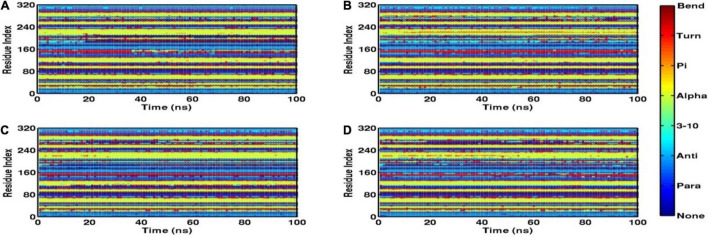
Time evolution plots of secondary structures using DSSP, **(A)** mtFabH **(B)** mtFabH-TLM, **(C)** mtFabH-C1, and **(D)** mtFabH-C2.

Root mean square fluctuation (RMSF) defines the average positional fluctuations of the protein residues from their initial position, which is important to determine the local dynamics of protein. It also provides an important clue about the contribution of amino acid residues involved in molecular interactions of protein–ligand for the spatial stability. In general, the residues belonging to stable secondary conformations (α-helix and β-sheet) show lower degree of residual fluctuations, whereas the residues belonging terminal (N-and C-terminal) and loop regions have a high degree of fluctuations. The RMSF plots of all four systems are shown in Figure 10. Results clearly display the remarkable dropdown in average atomic fluctuations of complex mtFabH-C1 as compared with remaining three systems, protein alone (mtFabH), mtFabH-TLM, and mtFabH-C2. Considering the results of RMSD and Rg, the higher average fluctuation of the residues for (mtFabH), mtFabH-TLM, and mtFabH-C2 prompted us to measure the time evolution plot of secondary structures. Results show no significant change in the secondary structure contents of all four systems. All the secondary conformations of α-helices and β-sheets were observed to be intact during the simulation. In fact, the time evolution plot of the contribution of H-bonds stabilizing the ligands at the active of mtFabH was observed to be consistent during the simulation. Figure 11 shows that the spatial binding of TLM stabilized through the maximum two H-bonds; however, the one H-bond observed infrequently appeared and disappeared during the simulation.

**FIGURE 12:**
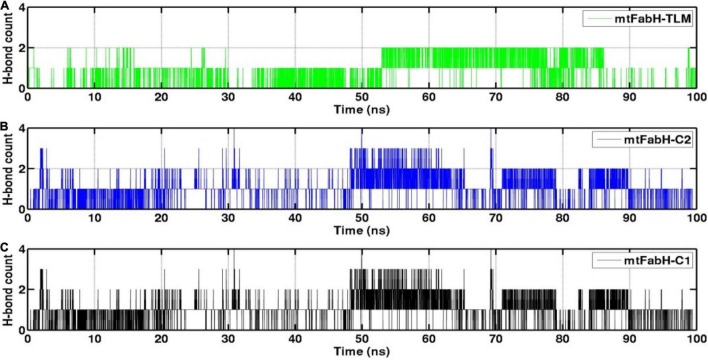
Representation of hydrogen bond (H-bond) count analysis of mtFabH with **(A)** inhibitor TLM, **(B)** lead compound C2 and **(C)** compound C1.

The H-bond interactions play a crucial role in the conformational stability of protein, its mobility, and molecular recognitions and interactions to perform the various biological functions. To understand the molecular binding of ligands, we also measured H-bond interactions with protein mtFabH using Amber tool cpptraj having a distance cutoff of 3.5Å and angle cutoff of 135° (Figure 12). Results show the maximum occupancy of two H-bonds between mtFabH and TLM. During the simulation, infrequent appearance of two H-bonds can be seen for initial 0–30 ns, which reappeared again at ∼55 ns, but it remains stable only up to ∼85 ns. The binding of lead compounds, C1 and C2, shows the maximum occupancy of three H-bonds; among them, only two H-bonds were observed to be consistent during the simulation.

### Essential Dynamics

**FIGURE 13:**
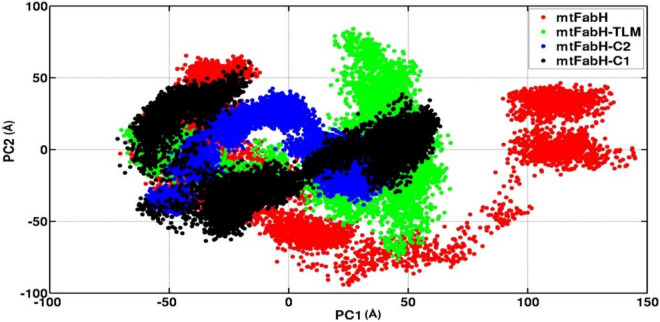
A comparative principal component analysis plots of mtFabH and mtFabH docked complexes with TLM and compounds C1 and C2.

We also investigated the collective motion of mtFabH and docked complexes with TLM, C1, and C2, applying the ED. ED is a standard statistical method, calculated by the projection of first two principal components (PCs), PC1 and PC2, which define the conformational space occupied by protein and protein–ligand systems during simulation. In Figure 13, the protein (mtFabH) alone explores a wide range of conformational space during simulation, which indicates the structural flexibility. However, all three docked complexes show that the collective motion was restricted to a confined conformational space, which suggests the binding of ligands results in the shifting of conformational space toward the stable equilibrium. The complex of mtFabH with compounds C1 and C2 shows a coherent change in the conformational dynamics, which is intended toward the stable equilibrium, whereas with small excursion the complex of mtFabH-TLM experiences the large conformational space as compared with complex with compounds C1 and C2. Thus, the ED analyses provide the elegance evidence of a more stable binding of compounds, C1 and C2, with mtFabH as compared with TLM.

### MM/PBSA (Free Binding Energy Estimation)

Finally, for the quantitative assessment of the binding affinity of ligands to mtFabH, we computed the free binding energy using MM/PBSA as shown in Table 4. The results show that binding of all three ligands, TLM, C1, and C2, is largely stabilized through the van der Waals (Δ*E*_vdW_) interactions: −26.43 ± 2.51, −31.79 ± 3.02, and −38.42 ± 2.94 kcal/mol, respectively. The collective contribution of electrostatic (Δ*E*_EEL_), the polar component of solvation free energy (Δ*E*_PB_), and the non-polar component of solvation free energy (Δ*E*_NPOLAR_), and the term accounts for solute–solvent dispersion interaction (Δ*E*_DISPERSION_) show the slightly more stable binding free energy of C2 (Δ*G*_*binding*_: −14.18 ± 3.06 kcal/mol) as compared with C1 (Δ*G*_binding_: −13.50 ± 3.25 kcal/mol), and TLM (Δ*G*_binding_: −9.21 ± 2.80 kcal/mol), respectively. Thus, the compound C2 can be explored as a potential lead molecule for the development of inhibitors against mtFabH in the therapy MTB.

**TABLE 4:** Binding free energy of three ligands against mtFabH, using PBSA.

	Δ*E*_vdW_	Δ*E*_EEL_	Δ*G*_PB_	Δ*G*_NPOL_	Δ*G*_DISP_	Δ*G*_TOTAL_
mtFabH-TLM	–26.43 ± 2.51	–3.03 ± 1.26	5.67 ± 1.21	–20.79 ± 1.15	35.38 ± 1.26	–9.21 ± 2.80
mtFabH-C2	–31.79 ± 3.02	–0.58 ± 0.23	3.69 ± 1.25	–22.13 ± 1.37	36.63 ± 1.64	–14.18 ± 3.06
mtFabH-C1	–38.42 ± 2.94	–5.79 ± 1.70	10.42 ± 1.39	–27.30 ± 1.43	47.59 ± 1.43	–13.50 ± 3.25

***ΔE**_vdW_: van der Waals component of molecular-mechanics force field energy of the solute.*

***ΔE**_EEL_: electrostatic component of molecular-mechanics force field energy of the solute.*

***ΔG**_PB_: polar component of solvation free energy of the solute.*

***ΔG_NPOL_**: non-polar component of solvation free energy of the solute.*

***ΔG_DISP_**: term that accounts for the solute-solvent dispersion interaction.*

## Conclusion

In the present study, KEGG-based biological network analysis indicates the involvement of mtFabH in the biosynthesis of mycolic acids, which is the key constituent of MTB cell wall. The virtual screening of antimycobacterial compounds from the ChEMBL library against the target protein results in the selection of 10 lead molecules. The molecular binding and pharmacokinetic profiles of lead molecules were compared with known mtFabH inhibitor, TLM, which suggests that compounds C1 and C2 may be explored as bioactive candidates for drug development. The molecular stabilities of ligand complexes were analyzed through the MD simulation, and the binding affinities of compounds were estimated by the free binding energy using MM/PBSA. MM/PBSA analysis indicates the higher binding affinity of compounds C2 as compared with C1 and TLM. Thus, the lead compound C2 may be explored as a potential candidate, which may contribute to the development of novel and potential inhibitors against mtFabH in the therapy MTB. However, further *in vitro* and *in vivo* authentications are warranted to establish their efficacy against mtFabH for the therapy of tuberculosis.

## Data Availability Statement

The raw data supporting the conclusions of this article will be made available by the authors, without undue reservation.

## Author Contributions

NK and RS performed the experiments. NK, RS, AB, AP, CM, and RM prepared and wrote the original draft. AP, AL, and M-SL were responsible for the supervision. NK, RS, AK, AB, MA-U-Z, RK, HS, RM, CM, and AL contributed to the data analysis and interpretation. All authors contributed to the review and approved the submitted manuscript.

## Conflict of Interest

The authors declare that the research was conducted in the absence of any commercial or financial relationships that could be construed as a potential conflict of interest.

## Publisher’s Note

All claims expressed in this article are solely those of the authors and do not necessarily represent those of their affiliated organizations, or those of the publisher, the editors and the reviewers. Any product that may be evaluated in this article, or claim that may be made by its manufacturer, is not guaranteed or endorsed by the publisher.
