# Supplementary material for: Identifying the Novel Inhibitors Against the Mycolic Acid Biosynthesis Pathway Target “mtFabH” of Mycobacterium tuberculosis
Source: Front Microbiol. 2022 May 6;13:818714. doi: 10.3389/fmicb.2022.818714 (PMC9121832; doi:10.3389/fmicb.2022.818714)
Supplement: Supplementary file 1 [file Data_Sheet_1.docx]

*Supplementary Information*

**Identify Promising Mycobacterium tuberculosis FabH Inhibitors**

Niranjan Kumar^1^, Rakesh Srivastava^1^, Raj Kumar Mongre^2,3^, Chandra Bhushan Mishra^4^, Amit Kumar^5,7^, Rosy Khatoon^6^, Atanu Banerjee^6^, Harpreet Singh^5^, Andrew. M. Lynn^1^, Myeong-Sok Lee^2^* and Amresh Prakash^7^*

^1^School of Computational & Integrative Sciences, Jawaharlal Nehru University, New Delhi-110067, India.

^2^ Molecular Cancer Biology Laboratory, Cellular Heterogeneity Research Center, Department of Biosystem, Sookmyung Women's University, Hyochangwon gil-52, Yongsan-Gu, Seoul 140-742, Republic of Korea.

^3^Department of Microbiology & Immunology, David H. Smith Center for Vaccine Biology and Immunology, University of Rochester Medical Center, 601 Elmwood Avenue, Box 609, Rochester, NY 14642.

^4^ Department of Pharmacology & Chemical Biology, Baylor College of Medicine, Baylor Plaza, Houston, Texas 77030.

^5^Indian Council of Medical Research - Computational Genomics Centre, All India Institute of Medical Research, New Delhi, India.

^6^Amity Institute of Biotechnology, Amity University, Haryana, Gurgaon- 122413, India.

^7^Amity Institute of Integrative Sciences and Health, Amity University, Haryana, Gurgaon- 122413, India.

**Address for Correspondence*

Amresh Prakash, PhD

Assistant Professor

Amity Institute of Integrative Sciences and Health (AIISH),

Amity University Haryana, Gurgaon-122413, India

Email: [amreshprakash@jnu.ac.in](mailto:amreshprakash@jnu.ac.in); [aprakash@ggn.amity.edu](mailto:aprakash@ggn.amity.edu)

Myeong-Sok Lee, Ph.D

Professor

Molecular Cancer Biology Laboratory, Cellular Heterogeneity Research Center, Department of Biosystem, Sookmyung Women's University, Hyochangwon gil-52, Yongsan-Gu, Seoul 140-742, Republic of Korea. Tel.: +8227109418

E-mail: [mslee@sookmyung.ac.kr](mailto:mslee@sookmyung.ac.kr)

**
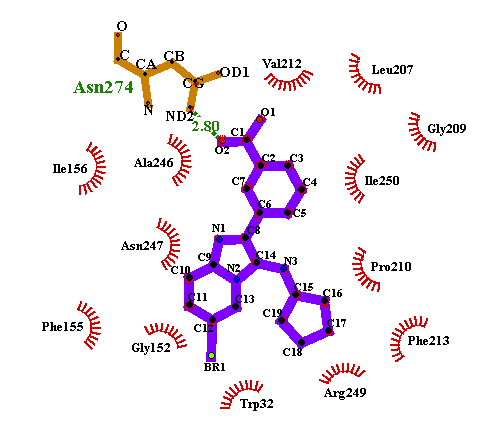
** **
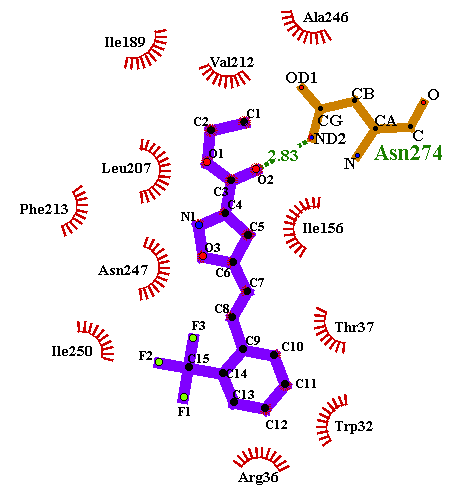
**

**CHEMBL549989 CHEMBL565500**

**
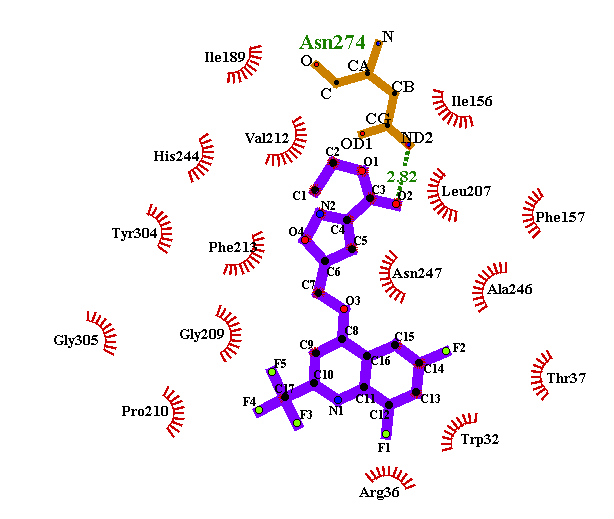

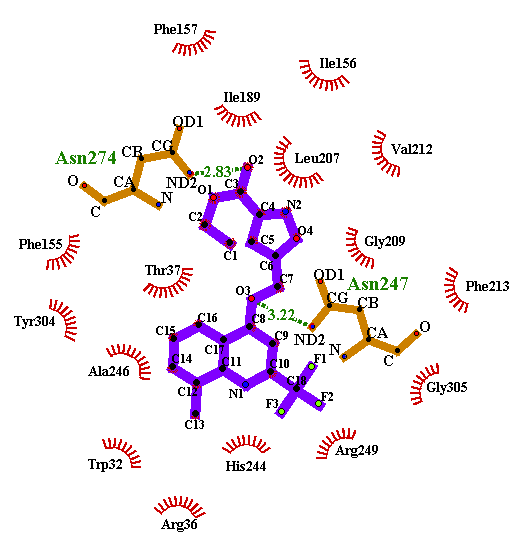
**

**CHEMBL515441 CHEMBL475041**

**
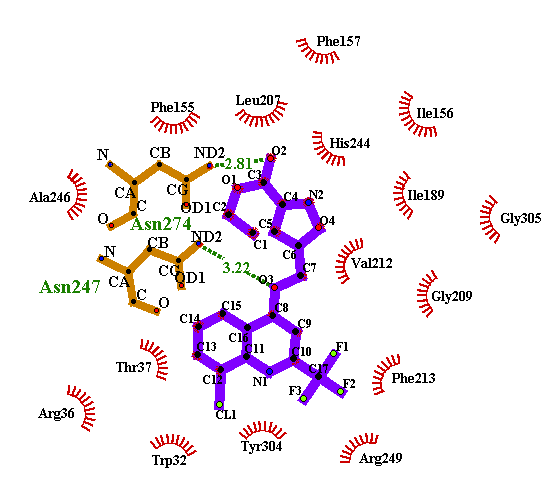

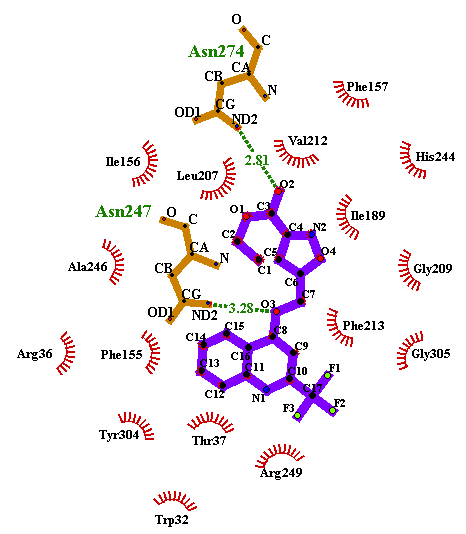
**

**CHEMBL474052 CHEMBL475851**

**
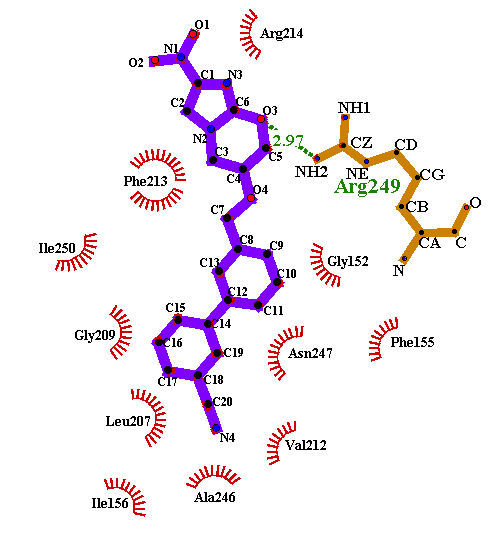
** **
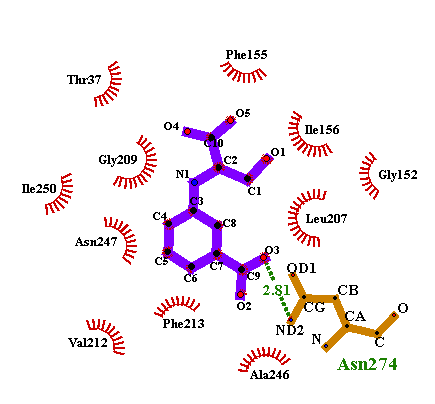
**

**CHEMBL572316 CHEMBL495223**

**Supplementary Figure S1:** LigPlot (2D) representation of top CHEMBL hit compounds**.**
